# Next Generation Sequencing and Comparative Genomic Analysis Reveal Extreme Plasticity of Two *Burkholderia glumae* Strains HN1 and HN2

**DOI:** 10.3390/pathogens11111265

**Published:** 2022-10-30

**Authors:** Sai Wang, Wenhan Nie, Ayizekeranmu Yiming, Peihong Wang, Yan Wu, Jin Huang, Iftikhar Ahmad, Gongyou Chen, Longbiao Guo, Bo Zhu

**Affiliations:** 1 Shanghai Yangtze River Delta Eco-Environmental Change and Management Observation and Research Station, Shanghai Cooperative Innovation Center for Modern Seed Industry, School of Agriculture and Biology, Shanghai Jiao Tong University, Shanghai 200240, China; 2 Department of Environmental Sciences, COMSATS University Islamabad, Vehari-Campus, Vehari 61100, Pakistan; 3 State Key Lab for Rice Biology, China National Rice Research Institute, Hangzhou 310006, China

**Keywords:** *Burkholderia glumae*, comparative genomics, genome annotation

## Abstract

*Burkholderia glumae* is an important rice pathogen, thus the genomic and evolutionary history may be helpful to control this notorious pathogen. Here, we present two complete genomes of the *B. glumae* strains HN1 and HN2, which were isolated from diseased rice seed in China. Average nucleotide identity (ANI) analysis shows greater than 99% similarity of the strains HN1 and HN2 with other published *B. glumae* genomes. Genomic annotation revealed that the genome of strain HN1 consists of five replicons (6,680,415 bp) with an overall G + C content of 68.06%, whereas the genome of strain HN2 comprises of three replicons (6,560,085 bp) with an overall G + C content of 68.34%. The genome of HN1 contains 5434 protein-coding genes, 351 pseudogenes, and 1 CRISPR, whereas the genome of HN2 encodes 5278 protein-coding genes, 357 pseudogenes, and 2 CRISPR. Both strains encode many pathogenic-associated genes (143 genes in HN1 vs. 141 genes in HN2). Moreover, comparative genomic analysis shows the extreme plasticity of *B. glumae*, which may contribute to its pathogenicity. In total, 259 single-copy genes were affected by positive selection. These genes may contribute to the adaption to different environments. Notably, six genes were characterized as virulence factors which may be an additional way to assist the pathogenicity of *B. glumae*.

## 1. Introduction

*Burkholderia glumae* (also known as *Pseudomonas glumae*), a gram-negative phytopathogenic bacterium, is the etiological agent of bacterial panicle blight (BPB) threatening rice cultivation worldwide [1]. This pathogen epiphytically grows on rice plants during the booting stage and multiplies on the surface of emerging panicles. Since 1956, when BPB was first documented in Japan, it has now been detected in more than 21 rice-producing countries [1,2,3]. It can reduce yields by up to 75% in severely infested fields, as it causes several types of damage, including grain abortion, floret sterility, and reduced milling quality [4,5,6,7,8,9,10]; for instance, it causes significant yield losses in the Mid-South USA in 1996, 2000 and 2010 [9,11,12]. The occurrence of BPB is highly dependent on weather conditions, such as prolonged high daily minimum temperatures and frequent rainfall during the rice-growing season, which contributes to the development of serious epidemics of BPB. The *B. glumae* shows relatively high growth at optimal temperature (30–35 °C) [13], therefore, it is considered that the disease may increase under the current scenario of global warming. A 1 °C raise in temperature can cause a 112 million $USD annual drop in consumer surplus in the US [10]. Moreover, none of the commercially available rice cultivars have adequate BPB resistance.

Earlier studies have demonstrated that the pathogenesis of *B. glumae* is a complex process involving several virulent factors. Several molecular genetics research groups have identified the major pathogenic determinants of *B. glumae*. Toxoflavin, a phytotoxin, is one of the most important virulence factors of *B. glumae* [14]. The genes responsible for toxoflavin biosynthesis and transport are encoded by the *toxABCDE* and *toxFGHI* operons, respectively [15,16]. Lipases are also important virulence factors, such as LipA and LipB [17,18,19], the former is an active extracellular lipase. LipB is involved in the biosynthesis of LipA and is required for the production of active LipA. It also has a significant impact on the stability of the proteins for proteolytic degradation. A substantial reduction in virulence was reported with the *B. glumae* strains deficient in flagella formation [20]. KatG catalase, the Hrp type-III secretion system (Hrp-T3SS), PehA and PehB polygalacturonase, lipopolysaccharide (LPS), and the Metalloprotease prtA are some of the additional factors responsible for the virulence of *B. glumae* [21,22,23,24,25]. The virulent factors of human and plant pathogens continue to evolve for successful infection in their respective hosts. *B. glumae* has the ability of cross-kingdom pathogenicity [17], thus, it is very important to study the evolution of *B. glumae* to find new virulence factors and provide candidate genes for disease resistance breeding. However, to accomplish this, we need to develop high-quality genomic resources. Therefore, in this study, we present two complete genomes of *B. glumae* that were isolated from diseased rice seeds in China. We annotate the protein-coding genes as well as repetitive elements and non-coding RNAs (ncRNAs). The population structure of *B. glumae* was analyzed. We believe that these two complete genomes of *B. glumae* will be a valuable resource for forthcoming research on this species and will facilitate studies on the control of this pathogen.

## 2. Materials and Methods

### 2.1. Strain Isolation, DNA Extraction, and Sequencing

*B. glumae* strains HN1 and HN2 were isolated from diseased rice seeds, obtained from Hunan Province, China, in 2016. A single colony of *B. glumae* strains HN1 and HN2 was inoculated into 2 mL of Luria-Bertani (LB) medium, and incubated for 24 h at 37 °C, and 170 rpm. Then, 1.5 mL of this culture was taken to isolate the genomic DNA by using a DNA purification kit (GeneJET Genomic DNA Purification Kit, Thermo, Waltham, MA, USA) as per the manufacturer’s instructions. Total genomic DNA was sequenced using the Oxford Nanopore platform (for genome assembly) and the Illumina NovaSeq platform (for genome surveying and base correction after assembly).

### 2.2. Genome Assembly and Annotation

The genome was assembled using raw Nanopore sequencing reads by Canu v1.8 [26] and circularized by Circlator v0.69-9 [27]. For 2 × 150 paired Illumina sequencing reads, low-quality sequences (Phred quality score < 30) were edited using TrimGalore v0.6.5 [28], then mapped to the assembled genome by bwa v0.7.17-r1188 [29] and the genome was corrected by Pilon v1.23 [30]. To learn more about plasmids in HN1 and HN2, we searched the sequences of plasmids against the NCBI nt database online. The genome completeness was assessed with BUSCO v5.1.2 [31] using the Burkholderiales data set, while genome annotation was performed by employing the Prokaryotic Genome Annotation Pipeline (PGAP) [32].

The orthologous groups of genes were annotated by eggNOG v5.0 [33]. The Gene Ontology (GO) terms for each gene were assigned using Blast2GO [34] software. To investigate the metabolic potential of *B. glumae*, KEGG Automatic Annotation Server (KAAS) [35] was used to get an overview of pathways. Protein domains were annotated by searching against the Pfam (34.0) database by using InterProScan (V 5.55-88.0) [36]. The annotation of carbohydrate-associated genes was performed using the dbCAN CAZyme domain HMM database, CAZY database [37], run from the dbCAN2 server [38]. The BLASTP v 2.10.1+ [39] search was performed to identify the virulence factor database (VFDB) [40] and the pathogen-host interactions database [41].

Genomic islands (clusters of genes of probable horizontal origin in bacterial or archaeal genomes) were predicted using the comparative genomics approach of IslandPick, plus the sequence composition-based approaches SIGI-HMM and IslandPath-DIMOB, as available in IslandViewer 4 [42]. When the regions appear in the results of at least one method, they were classified as genomic islands. Annotations were categorized as mobile genetic elements (MGEs) based on string matches to one of the following keywords: transposase, transposon, conjugative, integrase, integron, recombinase, conjugal, mobilization, recombination, plasmid, resolvase, relaxase and invertase [43]. Using HGTector v2.0b3, a homology/taxonomy technique was used to assess which genes were potentially acquired through horizontal gene transfer with the following parameters: —maxseqs 200—evalue 1 × 10 ^−20^ [44].

### 2.3. Comparative Analysis against Sequenced and Annotated B. glumae Genomes

A total of 25 strains including 24 *B. glumae* and 1 *B. gladioli* were collected from the NCBI database with annotation. The functions of the genes were annotated according to the methods described above. ANI analysis was performed to estimate the relatedness of the genomes by Pyani with the ANIb (based on BLAST) methods [45], and DNA–DNA hybridization (DDH) values of these genomes were predicted by the Genome-to-Genome Distance calculator 2.0, formula 2 [46]. Whole-genome alignments were generated for phylogenetic analysis through the mapping of DNA sequences against reference sequences. In this study, we used *B. glumae* ATCC 33617^T^ as a reference genome and this process was performed by SKA [47] with default settings. Recombinant regions were identified using Gubbins [48] and a recombination-masked alignment was generated with the post-processing script mask_gubbins_aln.py. Phandango [49] was used for visualizing Gubbins results with associated metadata. The resulting masked alignment was then utilized as input for generating a phylogenetic tree.

To identify OG (orthologous groups) of *B. glumae*, the amino acid sequences from the annotation were used in Orthofinder v2.5.2 [50]. Once all gene families had been clustered, we analyzed the pan-genome of *B. glumae.* We assigned the gene families that are present in all *B. glumae* strains in our dataset to the core genome. Unsurprisingly, most of them are involved in vital roles in bacterial survival as reported previously [51,52]. A shell genome is composed of the set of genes that are present in two or more strains but not all. Unique genes are those present in only one strain. Using the “micropan” R package [53], we next examined whether the pan-genome of *B. glumae* was open or closed. A rarefaction curve for the whole pan-genome was generated using 5000 permutations, each with a random genome input order. The curve was then fitted to the Heaps’ law model to establish the average number of unique ortholog clusters seen per genome and whether the pan-genome is open or closed [52]. Significantly, the alpha value represents the openness or closeness of the pan-genome, with a number less than 1 representing an open pan-genome and a value greater than 1 representing a closed pan-genome. The gene accumulation rarefaction curve was estimated by PanGP [54].

The phylogenetic relationships between the 26 *B. glumae* strains analyzed in this study were explored using single-copy genes. Single-copy gene sequences were extracted by a host python script and aligned by MAFFT v7.310 [55]. Low-quality alignments were trimmed by TrimAl v1.4.1 [56] using an “automated1” algorithm, and inferred a phylogenetic tree using IQ-TREE v2.1.3 [57], applying the model of best fit as assigned by ModelFinder [58]. The population structure was inferred by fastbaps [59] with the baps method.

### 2.4. Positive Selection and Functional Enrichment Analysis

Single-copy genes with fewer than four nonidentical sequences were excluded from the positive selection (PS) analyses. This minimum number of nonidentical sequences was due to the phylogenetic software (RAxML), as it requires four or more sequences to construct a phylogeny. The protein sequences in each OG were aligned using MUSCLE v3.8.31 [60] and the corresponding nucleotide sequence alignment was generated by PAL2NAL [61]. Phylogenies for each alignment were generated by RAxML v8.2.12 [62] using the GTR gamma substitution model. BUSTED (Branch-Site Unrestricted Statistical Test for Episodic Diversification) implemented in HyPhy v2.5.14 [63,64] was used to assess if a gene has experienced a positive selection at any site at the gene-wide level. By contrasting the constrained model (that is, disallowing for PS) with the unconstrained model (that is, allowing for PS), a likelihood ratio test was performed. The likelihood ratio test statistic was applied to determine the statistical significance. Genes were considered to have PS evidence if FDR-adjusted *p* < 0.05. GO and KEGG Pathway enrichment analysis was performed using the clusterProfiler R package [65].

## 3. Results

### 3.1. Genome Assembly and Properties

The genomes of *B. glumae* were sequenced using both long-read (Oxford Nanopore, Oxford, UK) and short-read technology (Illumina, San Diego, CA, USA). After quality-filtering, 103,577 long-reads with a mean length of 12,277 bp (Oxford Nanopore, Oxford, UK) and 4,963,807 paired-end Illumina reads (2 × 150 bp) were obtained for *B. glumae* HN1 assembly, whereas 47,188 long-reads with a mean length of 11,473 bp (Oxford Nanopore, Oxford, UK) and 5,493,123 paired-end Illumina reads (2 × 150 bp) were retained for *B. glumae* HN2 assembly. The assembled, closed genome of HN1 is 6,680,415 bp with an overall %G + C content of 68.06% and consists of five replicons; two chromosomes (chromosome1: 3,611,155 bp, %G + C 68.05; chromosome2: 2,869,204 bp, %G + C 68.57) and three plasmids (pBGHN1-1: 185,112 bp, %G + C 60.8; pBGHN1-2: 7606 bp, %G + C 63.29; pBGHN1-3: 7338 bp, %G + C 61.41). The genome of HN2 is 6,560,085 bp with an overall %G + C content of 68.34% and consists of three replicons; two chromosomes (chromosome1: 2,869,204 bp, %G + C 68.57; chromosome2: 2,745,532 bp, %G + C 68.99) and a single plasmid (pBGHN2: 281,656 bp, %G + C 62.17). The BLAST results of pBGHN1-2 and pBGHN1-3 show that all subject sequences are from the *Xanthomonas* genus, and the BLAST results of pBGHN1-1 and pBGHN2 show that all subject sequences are from the *Burkholderia glumae* species. BUSCO detected completeness of 99.4% (HN1) and 99.8% (HN2) with genome mode and 98.7% (HN1) and 98.8% (HN2) with proteins mode.

### 3.2. Genome Annotation

The overall genome statistics of HN1 and HN2 are detailed in Table 1 and Table S1. The strain HN1 contains 5434 protein-coding genes, 82 RNA genes, 351 pseudogenes, and 1 CRISPR, whereas strain HN2 contains 5278 protein-coding genes, 82 RNA genes, 357 pseudogenes, and 2 CRISPRs. Putative functions are assigned to 5045 (HN1) vs. 4975 (HN2), whereas 389 (HN1) vs. 303 (HN2) CDS are annotated as hypothetical proteins or proteins of unknown function.

### 3.3. Pathway Analysis

From the predicted protein-coding genes, 2925 (HN1) and 2912 (HN2) genes fall into 42 functional categories according to the Kyoto Encyclopedia of Genes and Genomes orthology in Table S1. Among these genes, 1795 (HN1) and 1767 (HN2) genes are connected to 245 and 247 different KEGG pathways. Across all categories, genes involved in carbohydrate metabolism (316 genes in both HN1 and HN2), amino acid metabolism (303 genes in HN1 vs. 302 genes in HN2), membrane transport (252 genes in HN1 vs. 246 genes in HN2), metabolism of cofactors and vitamins (223 genes in HN1 vs. 220 genes in HN2), signal transduction (184 genes in HN1 vs. 180 genes in HN2), energy metabolism (180 genes in HN1 vs. 181 genes in HN2), lipid metabolism (109 genes in HN1 vs. 105 genes in HN2) and xenobiotics biodegradation and metabolism (100 genes in HN1 vs. 102 genes in HN2) are the most abundant.

Additionally, several genes are affiliated with the cellular community—prokaryotes (213 genes in HN1 vs. 201 genes in HN2) and cell motility (110 genes in HN1 vs. 115 genes in HN2), which includes biofilm formation (126 genes in HN1 vs. 121 genes in HN2) pathway, quorum sensing pathway (101 genes in HN1 vs. 96 genes in HN2), flagellar assembly pathway (52 genes in both HN1 and HN2) and bacterial chemotaxis pathway (67 genes in HN1 vs. 62 genes in HN2). These pathways are essential for the pathogenesis of *B. glumae*. The genome of HN1 and HN2 carries genes from five types of secretion systems (I, II and VI), particularly type III (T3SS) and VI (T6SS), indicating that *B. glumae* has evolved an arsenal of genetic systems for environmental interaction.

### 3.4. EggNOG Functional Annotation

Furthermore, 4669 protein-coding genes in HN1 (4308 single EggNOG and 361 multi-EggNOG proteins) and 4615 protein-coding genes in HN2 (4253 single EggNOG and 362 multi-EggNOG proteins) are classified according to COG into 21 categories and 22 categories, respectively. In Table S2, we describe the EggNOG category distribution and frequency of functional annotation results. Over 18% of the proteins (987 proteins in HN1 vs. 936 proteins in HN2) have no known function, whereas 9% (469 proteins in HN1 vs. 456 proteins in HN2) are involved in transcription.

### 3.5. GO and Pfam Annotation

A total of 3964 genes in HN1 and 3912 genes in HN2 could be assigned to at least one GO category using the Blast2GO pipeline. Among them, 2725 genes in HN1 and 2689 genes in HN2 were classified in the biological process category, 355 genes in HN1 and 356 genes in HN2 were classified in the cellular component category, 884 genes in HN1 and 867 genes in HN2 were classified in the molecular function category (Table S3). There was a total of 31 functional GO terms annotated in this study. For each of the three main categories, the dominant GO terms were ‘cellular process’ (in ‘biological process’), ‘cellular anatomical entity’ (in ‘cellular component’), and ‘catalytic activity’ (in ‘molecular function’). In contrast, only a few genes were found for ‘carbon utilization’ (in ‘biological process’) and ‘toxin activity’ (in ‘molecular function’). In addition, ‘metabolic process’ (in ‘biological process’) also was abundant, and the ‘catalytic activity’ category only included two GO terms: ‘cellular anatomical entity’ and ‘protein-containing complex’. We also obtained the functional domains identified by interproscan, which revealed that 4565 genes in HN1 and 4531 genes in HN2 were annotated with different domains.

### 3.6. Enzymes Involved in Carbohydrate Metabolism

According to our results, HN1 and HN2 encode 1041 and 1035 CAZymes (Carbohydrate-Active Enzymes) genes, respectively. In enzyme annotation, three approaches were used: 123 genes (assigned by hmmer), 896 genes (assigned by eCAMI), and 219 genes (assigned by diamond) in HN1 whereas 892 genes (assigned by eCAMI), and 211 genes (assigned by diamond) in HN2. In HN1, these genes include 112 glycosyl transferase (GTs), 88 glycoside hydrolases (GHs), 14 carbohydrate esterases (CEs), and 9 auxiliary activities (AAs). In HN2, these genes include 111 glycosyl transferase (GTs), 81 glycoside hydrolases (GHs), 14 carbohydrate esterases (CEs), and 9 auxiliary activities (AAs).

### 3.7. Virulence Factor Annotation

To further understand the pathogenic potential of HN1 and HN2, we used blastp to search the VFDB and PHI databases for virulence genes. About 58 genes in HN1 and 56 genes in HN2 were assigned to virulence factors with the annotation of VFDB in Table S4. The genes identified as virulence factors were classified into seven categories: motility, immune modulation, effector delivery system, biofilm, adherence, regulation, and others. The genes involved in motility are the most prominent among these groups, with 37 genes in HN1 and 39 genes in HN2. PHI is a database that catalogs experimentally verified pathogenicity, virulence and effector genes from fungal, oomycete, and bacterial pathogens, which infect the animal, plant, fungal, and insect hosts. Eighty genes in HN1 and 77 genes in HN2 could be annotated with the PHI database. Knocking out these genes may result in the following seven results: reduced virulence, increased virulence (hypervirulence), lethal, loss of pathogenicity, effector (plant avirulence determinant), and unaffected pathogenicity. Knocking out 49 genes in HN1 and 47 genes in HN2 may result in lower pathogenicity. However, these two databases do not include the most important toxoflavin biosynthesis and transport clusters. So, we manually functionally annotated these pathogenic genes based on KEGG KO id. In total, 143 genes in HN1 and 141 genes in HN2 are associated with pathogenicity.

### 3.8. Genomic Island and Horizontal Gene Transfer

**Figure 1:**
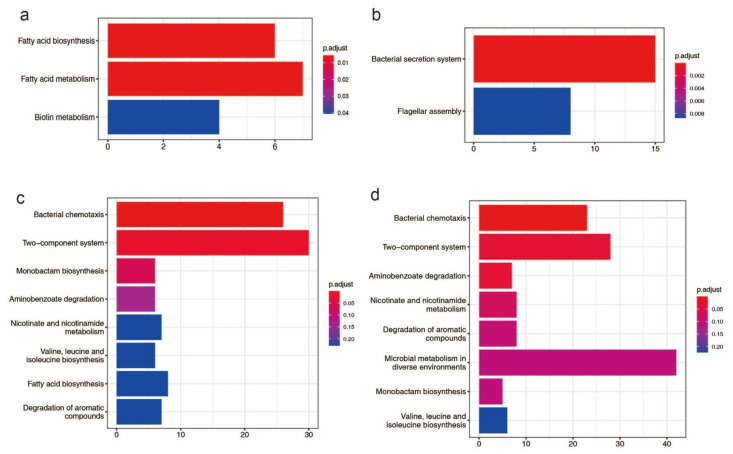
Bar plot KEGG enrichment analysis. Each bar in the figure represents a KEGG metabolic pathway, and the number of genes enriched in a pathway corresponds to the length of the bar. The degree of significance of the enrichment in a pathway is represented by p.adjust value. (**a**): KEGG enrichment analysis of genes found on genomic islands in HN1; (**b**): KEGG enrichment analysis of genes found on genomic islands in HN2; (**c**): KEGG enrichment analysis of potential horizontal transfer genes in HN1; (**d**): KEGG enrichment analysis of potential horizontal transfer genes in HN2.

There are 58 genomic islands (>750 kb) in the HN1 chromosome and 72 genomic islands (>786 kb) in the HN2 chromosome (Table S5). The length of the genomic island accounts for 11.58% of chromosome length in HN1 and 13.37% of chromosome length in HN2. These genomic islands include 573 genes in HN1 and 548 genes in HN2. Genes found on genomic islands perform a variety of functions such as transposase, integrase, transferase, transcriptional regulator, and type VI secretion system related proteins. For HN1, genes located on genomic islands were enriched for three KEGG pathways: fatty acid biosynthesis, fatty acid metabolism and biotin metabolism and for HN2, genes located on genomic islands were enriched for two KEGG pathways: bacterial secretion system and flagellar assembly (Figure 1a,b). For Gene Ontology (GO), these genes were enriched for DNA binding, cellular macromolecule metabolic process, DNA metabolic process and other terms, more details could be accessed in supplementary Figure S1.

663 genes of HN1 and 634 genes of HN2 were identified as potential horizontal transferable genes, which means that HN1 and HN2 may acquire these genes through horizontal transfer (Table S6). KEGG significant enrichment analysis showed these genes were mainly involved in bacterial chemotaxis, two-component system, monobactam biosynthesis, aminobenzoate degradation, nicotinate, and nicotinamide metabolism, valine, leucine and isoleucine biosynthesis, fatty acid biosynthesis, degradation of aromatic compounds, and microbial metabolism in diverse environments (Figure 1c,d). According to the GO significant enrichment analysis, these genes are mostly engaged in molecular function and biological processes. The GO enrichment results are shown in supplementary Figure S2.

### 3.9. Genome Comparison

**Figure 2:**
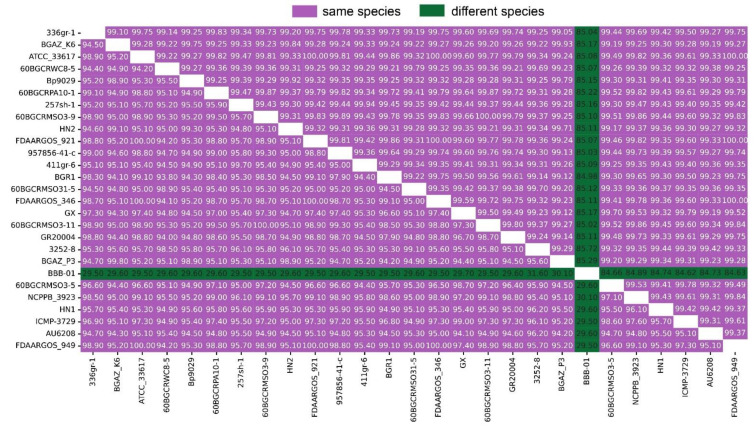
Matrix of average nucleotide identity (ANI) and DNA–DNA hybridization (DDH) values between 27 strains. The upper triangular matrix shows ANI values; the lower triangular matrix shows DDH values. ANI > 95% and DDH > 70% indicate the same species.

ANI and DDH have been used to compare bacterial genome sequences and is widely acknowledged as one of the most reliable measures of strain-relatedness. For the delimitation of bacterial species, an ANI value of 95% and DDH > 70% are recognized as the cutoff values. The ANI and DDH analysis result between HN1, HN2, other 24 strains of *B. glumae* and 1 *B. gladioli* collected from NCBI are detailed in Figure 2. The raw information of 27 strains is detailed in Table S7.

**Figure 3:**
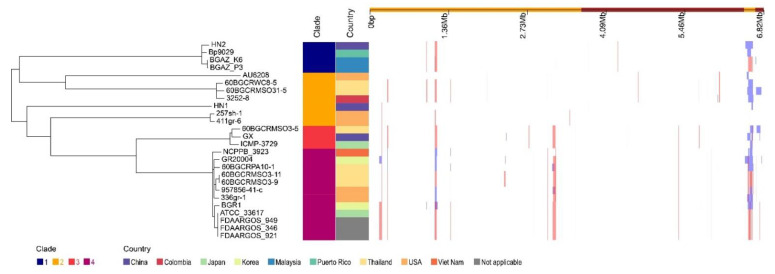
Phylogeography and recombination of *B. glumae*. Left, global phylogeny of *B. glumae*. The maximum likelihood tree constructed using the masked alignment of the whole-genome alignments. Middle, the metadata of the 26 strains. Four main population groups were identified using fastbaps and the country of strains. Right, recombination detected in *B. glumae*. The panel shows the chromosomal locations of the putative recombination events detected in each strain.

With the whole genome alignment as input, Gubbins predicts 157 recombination events in total (Figure 3). In total, 580 genes (543 protein-coding genes, 3 tRNA genes, and 34 pseudogenes) were detected in the recombination regions, and the total length of those genes is 431,358 bp (Table S8). Among the protein-coding genes, there are 37 phage-related proteins and 33 different types of transposases.

**Figure 4:**
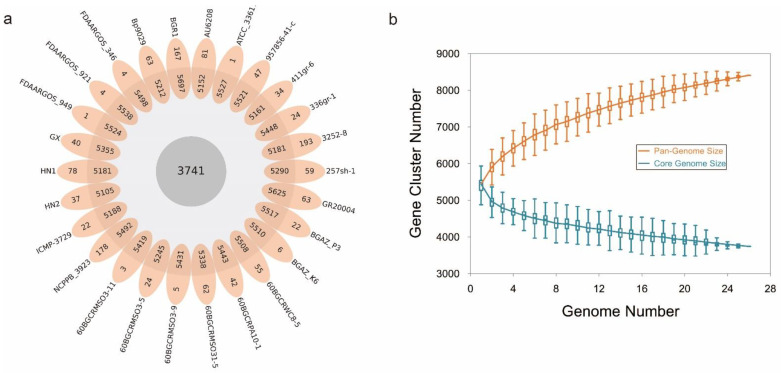
The pan-genome of *B. glumae*. (**a**): flower plot diagram showing core, shell and unique genes across all strains. The central circle shows the core genes number, the annulus shows the shell genes number and the petals show the unique genes number. (**b**): Pan and accessory (shell and unique) genome sizes as the number of genomes included increases.

**Figure 5:**
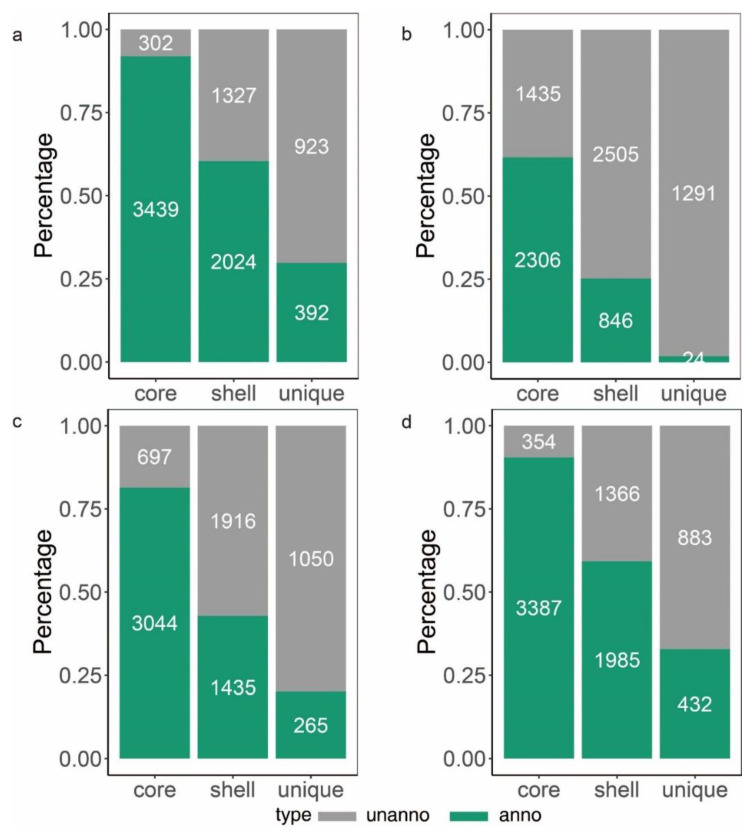
Number (indicated in the bars) and fraction of core, shell and unique genes. The green bar represents genes with annotations and the gray bar represents genes with no annotation. (**a**): the summary of genes annotated in EggNOG. (**b**): the summary of genes annotated in KEGG orthology. (**c**): the summary of genes annotated in GO orthology. (**d**): the summary of genes containing an annotated InterPro domain.

To learn more about how *B. glumae* gene families have evolved, we performed an OrthoFinder analysis using the proteins of 26 strains, and then those proteins were binned into 9391 orthologous groups (gene families). There were 3741 gene families conserved across these 26 *B. glumae* strains, including 2807 single-copy genes (just one ortholog in each gene family) (Figure 4a). The *B. glumae* pan-genome was composed of a core genome of 3741 orthologous groups in all 26 isolates, 4335 shell orthologous groups in (1 < shell < 26) of the isolates, and 1315 unique orthologous groups in only 1 of the isolates (Figure 3). The pan-genome was not closed, as seen by the core and pan plots since the number of pan-genes increased with the addition of approximately 26 genomes and the alpha value was 0.87, which is less than 1 (Figure 4b). A higher proportion of proteins with annotated functions were found in the core genome, more than 90% (3439) of the core orthologous groups were classified into categories compared with 45% (2024) of the shell orthologous groups and 30% (392) of the unique orthologous groups (Figure 5a). The same pattern could be found in the annotations of KEGG, GO, and pfam for three types of orthologous groups (Figure 5b–d).

**Figure 6:**
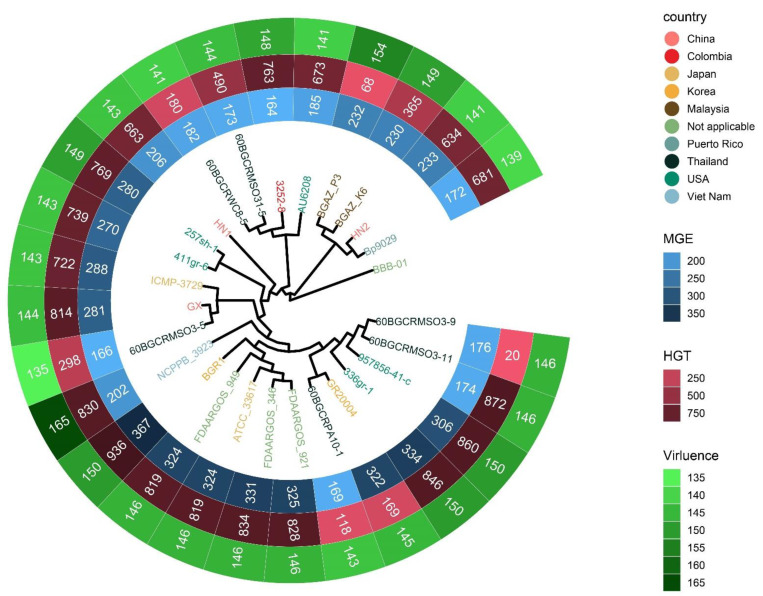
Phylogenetic tree detailing the country information of strains, the count of mobile genetic elements, potential horizontal transfer genes and virulence related genes. The tree was generated with IQTREE based on the alignment of single-copy gene sequences and visualized with ggtreeExtra R package. HN1 and HN2 are the genomes sequenced in this study. Strain names of 27 strains are color-coded by country.

To investigate the contents of shell and unique orthologous groups, mobile genetic elements and horizontal gene transfer events were accessed and shown in Figure 6, Tables S6 and S7. Hundreds of mobile genetic elements were found in each strain. For HN1, 181 mobile genetic elements were in chromosomes and 25 mobile genetic elements were in plasmids. For HN2, 187 mobile genetic elements were in chromosomes and 46 mobile genetic elements were in plasmids. These elements may contribute to the transfer of DNA between bacterial cells. Indeed, we did find many potential horizontal transfer genes. There are 663 and 634 potential horizontally-transferred genes in HN1 and HN2, respectively. And the potential donor TaxID is shown in the Table S6. To obtain deeper insight into the history and divergence times of *B. glumae,* the nucleotide sequences of all single-copy genes were concatenated (totaling 2,593,023 bp). We used IQTREE to compute a maximum likelihood tree from this alignment using the best model GTR + F + R2, which, as expected, provided a topology with highly supported nodes (Figure 6). Four main population groups were identified using fastbaps with this alignment (Figure 3, Table S7).

### 3.10. Positive Selection

**Figure 7:**
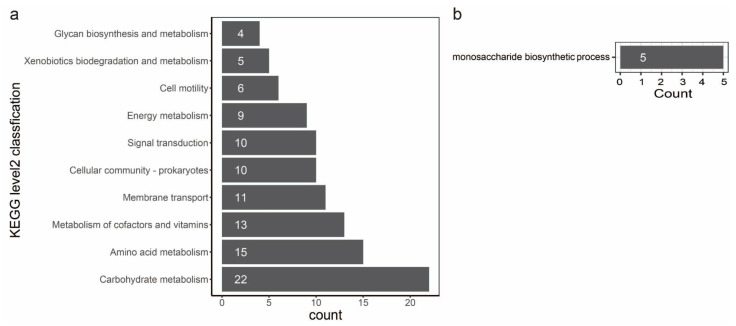
Bar plot of KEGG and GO analysis of orthologous groups that have positive selection. (**a**): the statistical analysis on top ten KEGG level2 classification. Each bar in the figure represents a KEGG level2 term, and the number of genes in a term corresponds to the length of the bar. (**b**): a statistical enrichment analysis on GO. Each bar in the figure represents a GO term, and the number of genes enriched in a term corresponds to the length of the bar.

In evolutionary biology, it is important to understand how natural selection affects genetic variation in populations. Alternatively, gene variation could be the primary force of adaptation to various environments. Therefore, we performed a gene-wide test for positive selection using a method called BUSTED [64]. This method detects genes that have had an elevated rate of nonsynonymous to synonymous substitutions at least one point in their recent evolutionary history. A total of 3777 orthologous groups were involved in the detection by BUSTED, and 259 orthologous groups have positive selection evidence (Table S9). To characterize the function of the genes evolving under positive selection, we annotated these genes against the different databases as described before (Table S9). Positive selection is active on genes directly engaged in fundamental metabolism and behavior, as seen by the repertoire of functions. The statistical analysis of the top ten KEGG level2 classification confirms this picture: we found orthologous groups affected by positive selection in different activities, such as carbohydrate metabolism, amino acid metabolism, metabolism of cofactors and vitamins, membrane transport, cellular community, signal transduction, energy metabolism, cell motility, xenobiotics biodegradation and metabolism and glycan biosynthesis and metabolism (Figure 7a). A statistical enrichment analysis of GO shows that five orthologous groups (OG0000311, OG0001169, OG0001550, OG0002093, and OG0003710) are significantly enriched in the monosaccharide biosynthetic process (Figure 7b, Table S9). Notably, six orthologous groups (OG0001644, OG0002397, OG0004213, OG0004298, OG0004319, and OG0004546) could be characterized as virulence factors (Table S9). Genes clustered in OG0004213 and OG0004298 are involved in toxoflavin biosynthesis and transport. Genes clustered in OG0001644 encode the flagellar basal body P-ring formation protein FlgA and are involved in the flagellar assembly pathway. Genes clustered in OG0002397 encode cyclic di-GMP phosphodiesterase CdpA and are involved in the biofilm formation pathway. Genes clustered in OG0002397 encode maltose alpha-D-glucosyltransferase and are involved in the starch and sucrose metabolism pathways. Genes clustered in OG0004546 encode a two-component system, chemotaxis family, and sensor kinase CheA are involved in bacterial chemotaxis.

## 4. Discussion

The ANI analysis values of *B. glumae* are greater than 99% and DDH analysis values of *B. glumae* are greater than 90% indicate that HN1 and HN2 belong to *B. glumae*. Interestingly, although the two strains were isolated from the same region, HN1 and HN2 did not share the strongest similarities. Note that the single-copy gene phylogenetic tree topology and population structure are also consistent with the ANI result, HN2 and HN1 are not clustered together. This demonstrates that there are multiple evolutionary routes of *B. glumae*. In addition, the strains that have clustered together are not always from the same country, which reveals that distinct strains may be diverse even if they originate from the same country.

A pan-genome is the whole set of nonredundant gene families from a taxonomically related group of species. An open pan-genome means that each new genome sequenced will provide novel genes. In this case, we have an open pan-genome. The number of pan-genes increased with the addition of genomes, and the alpha value of the Heaps’ law was 0.87, which is less than 1. So, we think that the pan-genome of *B. glumae* is open. In general, open pan-genomes have tiny core genomes and huge shell genomes. The core genome of *B. glumae* included 3741 orthologous groups, less than 40% of the whole pan-genome. In some bacterial species, genes from the core genome may be essential in pathogenicity and virulence [66,67]. Shell and unique genes are often acquired by horizontal gene transfer or evolved as a result of mutations in pre-existing genes. They are usually associated with a particular metabolism, pathogenicity, antibiotic resistance mechanism, or other environmental adaptation [51,68].

Bacterial genome plasticity enables the fluid exchange of DNA from one microorganism to another and may facilitate adaptation to different environmental conditions [69]. Horizontal gene transfer aids in the diversity and adaption of microorganisms, hence influencing genome plasticity. Horizontal gene transfer may result in substantial alterations to prokaryotic chromosomes. We found that 290 genes were in the recombination region of chromosome 1 with a total length of 203,026 bp and 37 genes were in the recombination region of chromosome 2 with a total length of 23,837 bp, and the functions of those genes are mainly related to phage, transposase, recombinase, and so on. Horizontal gene transfer through plasmids is a co-evolutionary mechanism [70], which is found in all complete assembly genomes of *B. glumae*. Genomic islands enable or have facilitated a significant portion of horizontal gene transfer [71]. The length of the genomic islands in HN1 and HN2 accounts for more than 10% of the chromosome, containing hundreds of mobile genetic elements and potential horizontal transfer genes. This is consistent with a previous comparative analysis between the genome of *B.glumae* strain BGR1 and the draft assembly of the *B. glumae* strain 336gr-1, which showed evidence of extensive genomic plasticity between genomes [72]. We also found hundreds of mobile genetic elements in each strain that could assist the exchange of DNA between bacterial cells. This evidence suggests the extreme plasticity of *B. glumae*.

The extreme plasticity of *B. glumae* also contributes to its pathogenicity. For HN1 and HN2, KEGG significant enrichment analysis showed these genes which could be acquired through horizontal transfer mainly involved in some pathways (Figure 1). Recent research shows that type VI secretion systems provide a functionally unique role in interspecies interactions and pathogenicity in *B. glumae* [73]. Bacterial flagellar motility and chemotaxis appear to be necessary for bacteria to effectively infect plant tissues [20]. In vivo transcriptional profiling of pathogenic *B. glumae* reveals that bacterial chemotaxis-mediated motility is one of the important infection processes [74]. In HN1, HJC54_RS20920, *toxF* (HJC54_RS01915, HJC54_RS08205), *toxA* (HJC54_RS08215), HJC54_RS04200, *toxB* (HJC54_RS08220) and *toxD* (HJC54_RS04200) are potential horizontal transfer genes, and *tofR* (HJC54_RS13625) is involved in the genomic island. In HN2, *fliM* (GAS18_RS09960), *fliN* (GAS18_RS09965), *fliO* (GAS18_RS09970), *fliP* (GAS18_RS09975), *fliQ* (GAS18_RS09980), *fliR* (GAS18_RS09985), *tofR* (GAS18_RS15890) and GAS18_RS10270 are involved in the genomic island, GAS18_RS13290, *toxD* (GAS18_RS18665), *toxB* (GAS18_RS18675), *toxA* (GAS18_RS18680), *toxF* (GAS18_RS18690, GAS18_RS24670) and GAS18_RS22355 are potential horizontal transfer genes. ToxR, a LysR-type regulator, regulates both the *toxABCDE* and *toxFGHI* operons in the presence of toxoflavin as a coinducer [15]. In our previous study, it has been demonstrated that a horizontally transferred gene is important for the full virulence of *B. glumae* [75]. So, it is inferred that the extreme plasticity of *B. glumae* may contribute to its pathogenicity.

Gene content variation could be the driver of adaptation to different environments. A positive selection study revealed that 259 single-copy genes were influenced by positive selection. Go enrichment analysis showed that five orthologous groups were significantly enriched in the monosaccharide biosynthetic process. Among them, genes clustered in OG0000311 encode 2-dehydro-3-deoxyphosphooctonate aldolase and are involved in the lipopolysaccharide (LPS) biosynthesis pathway. The LPS is the fundamental constituent of the outer membrane in gram-negative bacteria. The previous investigation found that the LPS, specifically the core OS region, is necessary for *B. glumae* resistance to environmental stress and complete pathogenicity [21]. In addition, six virulence factors that were under positive selection. These genes affected by positive selection might be associated with their adaption to various environments and evaded immune recognition.

Overall, we assembled two complete genomes derived from novel isolates of the species *B. glumae* (HN1 and HN2) using Illumina and Oxford Nanopore technologies. Gene function annotation indicates that HN1 and HN2 may be fully pathogenic. Comparative genomic analysis reveals the extreme plasticity of *B. glumae*, which may play a key role in its pathogenicity. Notably, six virulence factors were under positive selection, which may be another way of ensuring its pathogenicity of *B. glumae.* The discovery of this new genomic resource is expected to benefit the improvement of our knowledge of this species as well as ongoing work to create bacterial panicle blight control strategies.

## Figures and Tables

**Table 1:** Comparison of Assembly and Annotation Statistics for the *Burkholderia glumae* HN1 and HN2 genomes.

	HN1	HN2
Status	Complete	Complete
Genome size (bp)	6,680,415	6,560,085
GC content (%)	68.06	68.34
DNA replicons	5	3
Total genes	5867	5717
Protein-coding genes	5434	5278
RNA genes	82	82
rRNA genes	15	15
tRNA genes	63	63
ncRNA	4	4
Pseudogenes	351	357
CRISPR	1	2
Pathogenic associated genes	195	121
Genes with function prediction	5045	4975
Genes connected to KEGG pathways	2487	2477
Genes connected to KEGG Orthology (KO)	2955	2930
Genes assigned to COGs	4604	4545
Genes assigned to GOs	3420	2812

## Data Availability

The sequence assembly and annotations can be found at the National Center for Biotechnology Information (NCBI) under Bioproject PRJNA576729. *Burkholderia glumae* HN2 assembly is available on GenBank under accession number CP052864- CP052868. *Burkholderia glumae* HN2 assembly is available on GenBank under accession number CP052132-CP052134.

## Supplementary Materials

The following supporting information can be downloaded at: https://www.mdpi.com/article/10.3390/pathogens11111265/s1, Figure S1: Bar plot GO enrichment analysis; Figure S2: Bar plot GO enrichment analysis; Table S1: KEGG annotation statistics of *Burkholderia glumae* strains HN1 and HN2; Table S2: COG annotation statistics of *Burkholderia glumae* strains HN1 and HN2; Table S3: GO annotation statistics of *Burkholderia glumae* strains HN1 and HN2; Table S4: Genome annotation of *Burkholderia glumae* strains HN1; Table S5: Genomic islands information of *Burkholderia glumae* strain HN1; Table S6: Potential HGTs of 18 strains; Table S7: General information of 27 strains; Table S8: The annotation of genes located in recombined regions of ATCC 33617; Table S9: The annotation of single copy genes under positive selection.
