# Supplementary material for: Next Generation Sequencing and Comparative Genomic Analysis Reveal Extreme Plasticity of Two Burkholderia glumae Strains HN1 and HN2
Source: Pathogens. 2022 Oct 30;11(11):1265. doi: 10.3390/pathogens11111265 (PMC9698995; doi:10.3390/pathogens11111265)
Supplement: Supplementary file 1 [file pathogens-11-01265-s001.zip › Figure S2 HGT go enrich.pdf]

a

b

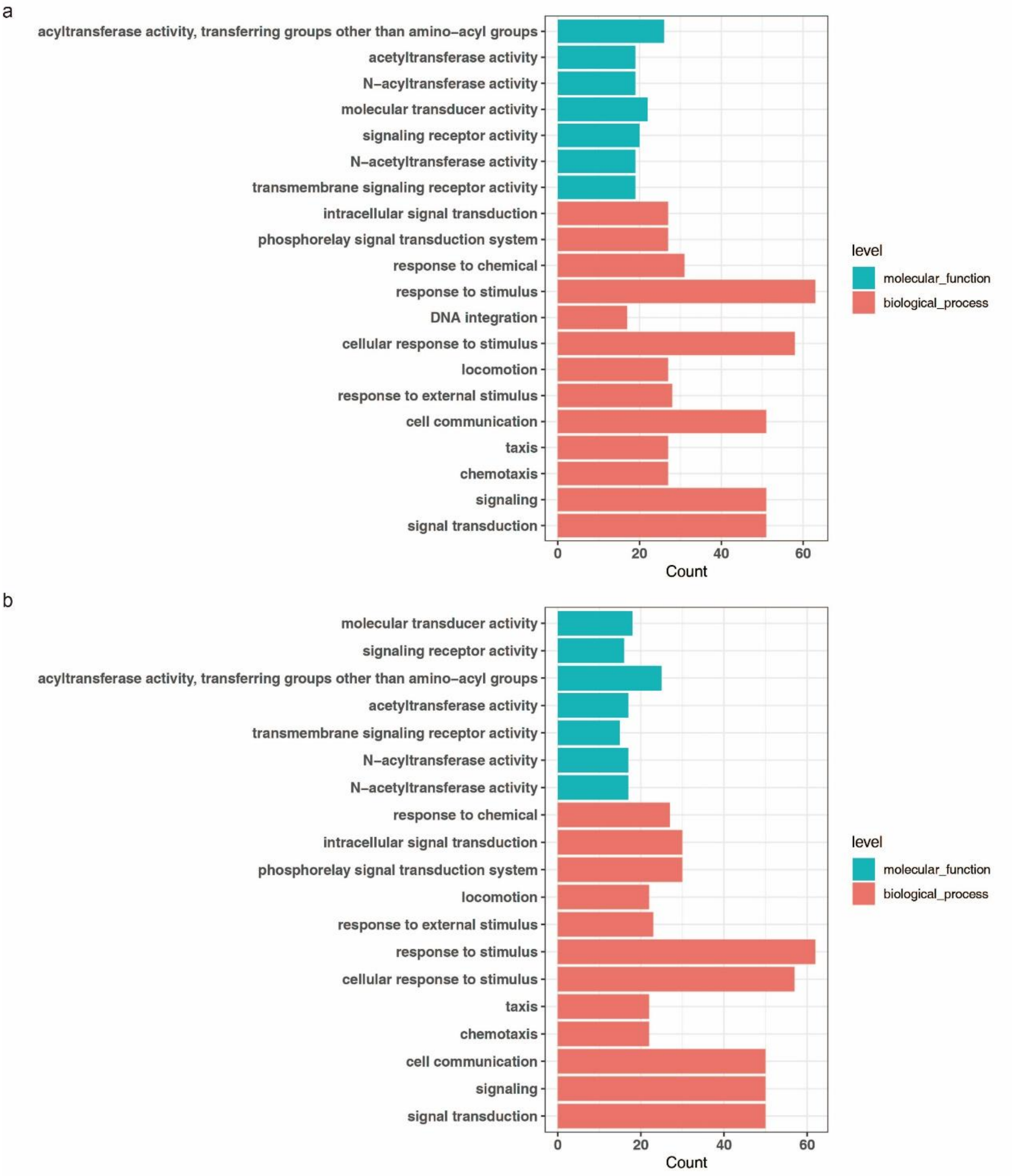

Figure S2: Bar plot GO enrichment analysis. Each bar in the figure represents a GO term, and the number of genes enriched in a term corresponds to the length of the bar. The degree of significance of the enrichment in a term is represented by p.adjust value. a: GO enrichment analysis of potential horizontal transfer genes in HN1; b: GO enrichment analysis of potential horizontal transfer genes in HN2.
